# Individualized theta transcranial alternating current stimulation modulates mnemonic discrimination and event-related potential responses to positive information in individuals with elevated depressive symptoms

**DOI:** 10.1093/scan/nsag079

**Published:** 2026-10-16

**Authors:** Xinyu Huang, Min Zhou, Chun Wang, Yuxin Huang, Wenchang Wang, Jianfei Shao, Junyu Mao, Jiaqi Li, Lei Zhang, Rong Ye, Chunyan Zhu, Yanghua Tian, Kongliang He, Fengqiong Yu, Kai Wang

**Affiliations:** School of Mental Health and Psychological Sciences, Anhui Medical University, Hefei, 230032, China; Department of Psychology, Faculty of Social Science, University of Macau, Macau, China; School of Mental Health and Psychological Sciences, Anhui Medical University, Hefei, 230032, China; School of Mental Health and Psychological Sciences, Anhui Medical University, Hefei, 230032, China; School of Mental Health and Psychological Sciences, Anhui Medical University, Hefei, 230032, China; School of Mental Health and Psychological Sciences, Anhui Medical University, Hefei, 230032, China; School of Mental Health and Psychological Sciences, Anhui Medical University, Hefei, 230032, China; School of Mental Health and Psychological Sciences, Anhui Medical University, Hefei, 230032, China; School of Mental Health and Psychological Sciences, Anhui Medical University, Hefei, 230032, China; School of Mental Health and Psychological Sciences, Anhui Medical University, Hefei, 230032, China; School of Mental Health and Psychological Sciences, Anhui Medical University, Hefei, 230032, China; School of Mental Health and Psychological Sciences, Anhui Medical University, Hefei, 230032, China; School of Mental Health and Psychological Sciences, Anhui Medical University, Hefei, 230032, China; School of Mental Health and Psychological Sciences, Anhui Medical University, Hefei, 230032, China; Anhui Mental Health Center, Hefei Fourth People’s Hospital, Anhui Medical University Affiliated Psychological Hospital, Hefei, 230022, China; School of Mental Health and Psychological Sciences, Anhui Medical University, Hefei, 230032, China; School of Mental Health and Psychological Sciences, Anhui Medical University, Hefei, 230032, China; Anhui Province Key Laboratory of Cognition and Neuropsychiatric Disorders, Anhui Medical University, Hefei, 230032, China; Collaborative Innovation Center of Neuropsychiatric Disorders and Mental Health, Anhui Medical University, Hefei, 230032, China; The First Affiliated Hospital of Anhui Medical University, Anhui Medical University, Hefei, 230022, China

**Keywords:** mnemonic discrimination, pattern separation, individualized protocol, transcranial alternating current stimulation , event-related potentials

## Abstract

Individuals with elevated depressive symptoms often show impairments in positive memory processing. This randomized, double-blind study investigated whether individualized theta-frequency transcranial alternating current stimulation (tACS) using an orbitofrontal cortex (OFC)-oriented montage modulates mnemonic discrimination and ERP responses to positive information. Sixty-one individuals with elevated depressive symptoms received either real or sham stimulation before completing an emotional mnemonic discrimination task. Behavioral results showed that lure discrimination increased following real stimulation but not sham stimulation. EEG results showed that real stimulation modulated ERP responses during positive memory encoding and retrieval, as reflected by increased late positive potential amplitudes during encoding and altered early retrieval-related ERP amplitudes. These findings suggest that individualized theta-tACS may influence both mnemonic discrimination performance and neural responses to positive information.

Highlights

- Individualized theta-tACS delivered with an OFC-oriented montage improved lure discrimination in individuals with elevated depressive symptoms.
- Theta-tACS modulated LPP amplitudes during positive memory encoding.
- Theta-tACS selectively modulated early, but not late, retrieval-related ERP amplitudes as a function of emotional valence.
- Behavioral and ERP findings suggest complementary effects of theta-tACS on mnemonic discrimination and neural responses to positive information.

## Introduction

Depression significantly impairs daily functioning and quality of life (Gao *et al*. 2020). Of particular relevance is subclinical depression (SD), characterized by depressive symptoms that do not meet the diagnostic threshold for major depressive disorder (MDD) (Cuijpers and Smit 2004). SD has been identified as an important risk factor for the subsequent development of MDD (Fogel *et al*. 2006, Lee *et al*. 2019). Investigating the cognitive and neural mechanisms underlying SD may therefore provide important insights into early intervention and prevention strategies for depression. Episodic memory deficits have increasingly been recognized as a prominent cognitive feature of depression. Research indicates that both clinically and subclinically depressed individuals exhibit impairments in the recall of positive and neutral episodic memories, whereas memory for negative information appears relatively preserved (James *et al*. 2021). Notably, these individuals lack the positive memory bias observed in healthy populations (Everaert *et al*. 2022). Although activating positive memories has been shown to reduce depressive-like behaviors and promote emotional resilience (Ramirez *et al*. 2015), most human interventions have focused primarily on reducing negative affect rather than enhancing the processing of positive information (Vanderlind *et al*. 2020). A key behavioral manifestation of this impairment is overgeneralized autobiographical memory recall (e.g. recalling broad categories rather than specific events), which correlates with the severity of depression. Such overgeneralization may reflect impairments in pattern separation, a fundamental memory process that enables the formation of distinct representations for highly similar experiences. Pattern separation is a fundamental memory process that reduces interference between similar experiences by generating distinct memory representations, thereby supporting accurate episodic memory retrieval (Norman 2010, Yassa and Stark 2011). Individuals at risk for depression have been shown to overgeneralize ambiguous stimuli as negative (Dearing and Gotlib 2009), suggesting potential impairments in pattern separation. Supporting this, Shelton and Kirwan (2013) found that higher depression severity was associated with poorer performance on tasks measuring pattern separation. However, as Dillon and Pizzagalli (2018) noted, most studies have only examined pattern separation for neutral and negative information. Consequently, it remains unclear whether pattern separation deficits extend to the processing of positive episodic memories. Given the important role of positive memories in emotional well-being, enhancing mnemonic discrimination related to positive information may represent a promising target for improving depressive symptoms. Therefore, investigating mnemonic discrimination for positive information may provide important insights into both the cognitive mechanisms of depression and the development of novel intervention strategies.

Mnemonic discrimination is supported by distributed memory-related networks rather than by the hippocampus alone. Although hippocampal CA3/dentate gyrus mechanisms are central to pattern separation (Yassa and Stark 2011), successful discrimination of similar experiences also requires contextual, affective, and goal-relevant information to be integrated with memory representations. The OFC is a key prefrontal node within this broader network. It is anatomically and functionally connected with medial temporal lobe structures, including the hippocampus and amygdala, and may contribute to mnemonic discrimination by integrating contextual and value-related information to guide flexible memory-based decisions (Young and Shapiro 2011, Wikenheiser and Schoenbaum 2016, Knudsen and Wallis 2022, Zheng *et al*. 2022, Costa *et al*. 2023, Qiu *et al*. 2024). Thus, rather than serving as an isolated stimulation target, the OFC may provide a prefrontal entry point for influencing memory-related hippocampal—amygdala—prefrontal network dynamics.

Theta oscillations (4–8 Hz) have been consistently implicated in episodic memory encoding, retrieval, associative binding, and emotional pattern separation, while previous theta-tACS studies have demonstrated modulation of memory-related performance (Clouter *et al*. 2017, Zheng *et al*. 2019, Shtoots *et al*. 2024). Individualizing the stimulation frequency according to each participant’s intrinsic theta peak was intended to reduce the mismatch between externally applied stimulation and endogenous neural oscillations, thereby increasing the likelihood of resonance or entrainment within memory-related networks (Kasten *et al*. 2019, Reinhart and Nguyen 2019, Zhang *et al*. 2022). These findings provided the theoretical and empirical rationale for selecting individualized theta-frequency stimulation in the present study.

High-definition transcranial alternating current stimulation (HD-tACS) is a well-established non-invasive neuromodulation technique capable of modulating theta-band oscillations. It operates by entraining (phase-locking) and resonating with endogenous neural oscillations through sinusoidal currents (Ali *et al.* 2013). HD-tACS has been shown to induce lasting neuroplastic changes while maintaining a favorable safety profile (Kar and Krekelberg 2012, Clancy *et al*. 2018). Its potential is evidenced by studies showing that alpha-tACS can produce lasting improvements in depressive symptoms (Alexander *et al*. 2019), and that prefrontal theta-tACS can enhance episodic memory retrieval (Shtoots *et al*. 2024). Given the established role of theta oscillations in episodic memory and emotional pattern separation, theta-frequency stimulation may represent a particularly promising approach for modulating memory-related neural networks. However, despite growing evidence for the cognitive benefits of theta-frequency stimulation, the effects of theta-tACS on mnemonic discrimination and neural responses to positive information remain largely unexplored.

A central feature of the present intervention was the use of individualized theta-frequency stimulation. Conventional fixed-frequency tACS protocols apply the same stimulation frequency to all participants, but intrinsic theta rhythms vary across individuals. This inter-individual variability may reduce the consistency of tACS effects when a fixed frequency is used. Individualizing the stimulation frequency to each participant’s intrinsic theta frequency may reduce frequency mismatch between the external alternating current and endogenous oscillatory activity, thereby increasing the likelihood of resonance or entrainment with the targeted memory-related network (Miniussi *et al*. 2013, Kasten *et al*. 2019, Zhang *et al*. 2022). From this perspective, individualized theta-tACS represents an EEG-informed neuromodulation strategy designed to align stimulation parameters with each participant’s endogenous oscillatory profile. In the present study, we therefore used individualized theta-frequency stimulation delivered with an OFC-oriented montage to examine whether this individualized intervention could modulate mnemonic discrimination and ERP responses to positive information in individuals with elevated depressive symptoms.

Event-related potentials (ERPs) provide high temporal resolution for examining memory encoding and retrieval. During encoding, we examined the late positive potential (LPP), a centro-parietal component associated with emotional memory encoding and motivational salience (Cuthbert *et al*. 2000). During retrieval, we assessed ERP differences between correctly recognized targets and correctly rejected lures. Although the old/new effect is a contrast-based measure rather than a canonical ERP component, it can be evaluated using condition-specific amplitudes with trial type included as a within-subject factor in a factorial ANOVA (Curran *et al*. 2006, Wolff *et al*. 2014, Chow *et al*. 2021). Early and late old/new effects have been associated with earlier recognition—or familiarity-related processes and later recollection—or post-retrieval evaluative processes, respectively (Yu and Rugg 2010, Weymar *et al*. 2011, Leynes and Nagovsky 2016). These measures were used to assess whether individualized theta-tACS modulated encoding- and retrieval-related neural activity.

This study aimed to investigate whether individualized theta-frequency tACS delivered with an OFC-oriented montage could modulate mnemonic discrimination and ERP responses to positive information in individuals with elevated depressive symptoms. Mnemonic discrimination was assessed using an emotional mnemonic discrimination task (MDT). Behavioral discrimination performance was quantified using the Lure Discrimination Index (LDI), which corrects for general response bias toward “new” responses. We hypothesized that individualized theta-tACS would improve mnemonic discrimination, as reflected by increased LDI scores in the real stimulation group relative to the sham group. We further hypothesized that theta-tACS would modulate ERP indices associated with positive memory encoding and retrieval, including the LPP and the early and late old/new effects. In addition, exploratory trial-level analyses were conducted to examine whether ERP activity predicted successful lure discrimination performance.

## Materials and methods

### Participants

The minimum sample size for an effect size of 0.35 (ηp² = 0.11) based on previous research (Clancy *et al*. 2018), a statistical test power of 0.95, and a significance level of 0.05 calculated using G*Power 3.1 (Faul *et al*. 2009) was 30. Additional participants were recruited to allow for attrition and data exclusion. Eligible participants had a Beck Depression Inventory-II (BDI-II) score ≥13, were right-handed, and had normal or corrected-to-normal vision. Exclusion criteria included unstable medical conditions, pregnancy or breastfeeding, substance use disorder, neurological or diagnosed psychiatric disorders, and brain stimulation within the preceding 2 months. All participants provided written informed consent and received RMB 200 upon completion. Sixty-one participants were assigned to the real stimulation group (*n* = 33; 24 females; age = 20.49 ± 1.62 years) or the sham group (*n* = 28; 18 females; age = 20.46 ± 1.47 years). Four participants were excluded from EEG analyses because of excessive artifacts (*n* = 3, real group) or corrupted data (*n* = 1, sham group), yielding a final EEG sample of 57 participants (real: *n* = 30; sham: *n* = 27).

### Experimental design and task

The randomized, double-blind, sham-controlled pre-post design is shown in Fig. 1. Participants were assigned to real or sham tACS using a predefined randomization list, with allocation and stimulation configuration performed by a research assistant uninvolved in EEG recording or behavioral assessment. On Day 1, participants completed demographic and questionnaire assessments, a 5-min eyes-open resting-state EEG recording for estimating individualized theta frequency (ITF), and the pre-stimulation emotional MDT with concurrent EEG. On Day 2, participants received 30 min of individualized theta-frequency tACS or sham stimulation. Immediately after stimulation, participants completed the post-stimulation MDT while EEG was recorded, using a stimulus set different from that used on Day 1. Questionnaire assessments and stimulation-related manipulation checks were administered as described in the Questionnaires and Manipulation Checks section.

**Figure 1:**
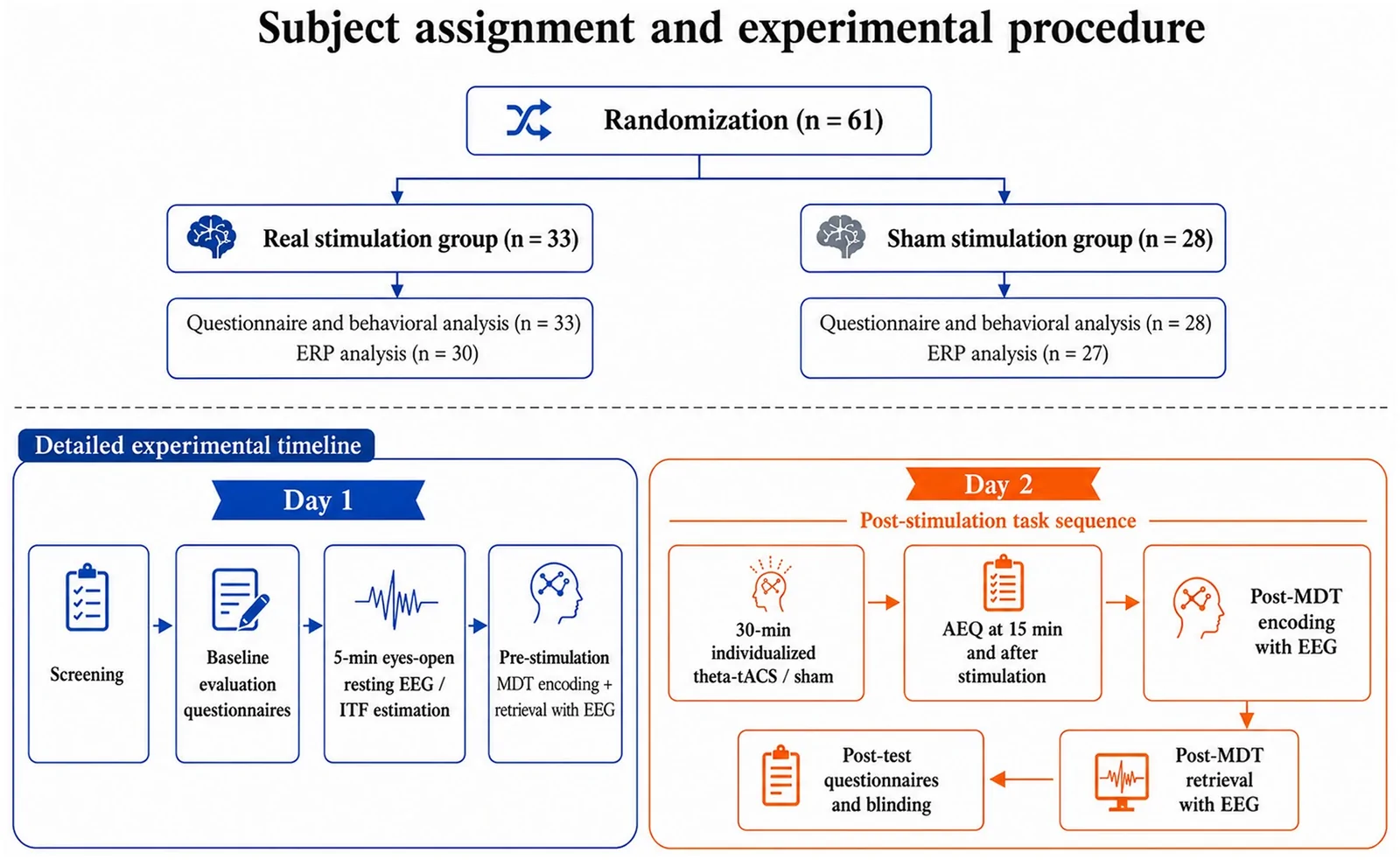
Diagram illustrating the subject assignment and experimental procedure. tACS, transcranial alternating current stimulation; ERP, event-related potential; AEQ, adverse effects questionnaire; ITF, individualized theta frequency.

The MDT was programmed using E-Prime 2.0 (Schneider 2012) and comprised encoding and retrieval phases (Fig. 2). During encoding, a fixation cross (1000–1500 ms) preceded a neutral or positive image (2000 ms), which participants rated on a 9-point valence scale. The retrieval phase followed a similar trial structure; however, instead of rating emotional content, participants classified each image as either “old” or “new.” The retrieval phase included three types of images for both neutral and positive conditions: “target” (an image previously shown during encoding), “lure” (an image similar to a previously shown one), and “foil” (a completely novel image not presented before).

**Figure 2:**
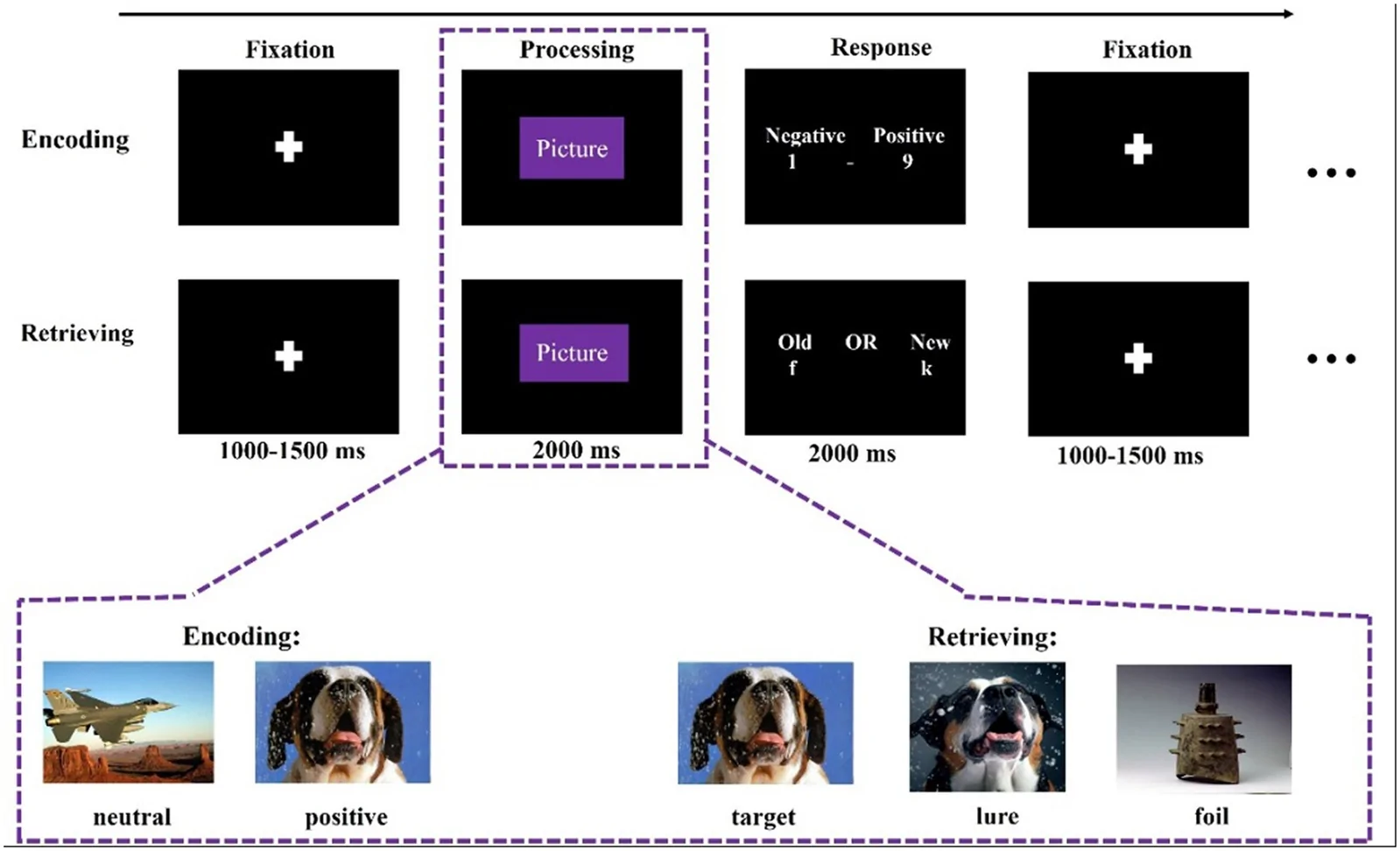
Mnemonic discrimination task (MDT) diagram. During the encoding phase, participants viewed images for 2000 ms preceded by a fixation (1000–1500 ms), and rated the emotional valence of each image using keys 1–9 (1 = very negative, 9 = very positive). During the retrieval phase, participants were shown target (previously shown during encoding), lure (visually similar to those shown), and foil (novel items) images and asked to classify them as “old” or “new” within 2000 ms.

Two stimulus sets (A and B) were assigned to the pre- and post-tests using an ABBA counterbalancing order. Target–lure similarity was validated in an independent sample of 31 college students and controlled within each valence condition because emotional images may be perceived as more similar than neutral images (Zheng *et al*. 2019). The sets were matched for target–lure similarity, valence, arousal, salience, and visual attributes, with no significant set-related effects or interactions (all *P* > .05; Tables S1–S3, see online supplementary material for a color version of these tables). Visual attributes were computed following Marchewka *et al*. (2014). Stimuli included 197 neutral and 200 positive images from the Chinese Affective Picture System (Lu *et al*. 2005), supplemented by 52 Midjourney-generated images to provide sufficient lures. Detailed validation procedures, statistical results, and stimulus composition are reported in the Supplementary Methods.

### Stimulation protocol

The current protocol was delivered using a Soterix Medical MXN-9 High-Definition Transcranial Electrical Stimulator (Soterix Medical, New York, NY, USA). Participants were asked to wear a customized BrainCap (Brain Vision) embedded with eight 12-mm-diameter Ag-AgCl electrodes. Electrode placement and stimulation intensity followed a previously established protocol (Grover *et al*. 2021). Specifically, tACS was applied through eight electrodes positioned at Ex17 (−0.6 mA), Ex18 (−0.6 mA), Fp1 (−0.3 mA), Fp2 (−0.3 mA), Cz (0.6 mA), C5 (0.3 mA), C6 (0.3 mA), and Iz (0.6 mA), delivering a ±1.8 mA (peak-to-peak) bidirectional sinusoidal current through the OFC-oriented montage. Electric-field modeling using SimNIBS (Saturnino *et al*. 2019) suggested relatively higher field magnitude in bilateral orbitofrontal/ventromedial prefrontal regions for the current OFC-oriented montage (see Fig. 3).

**Figure 3:**
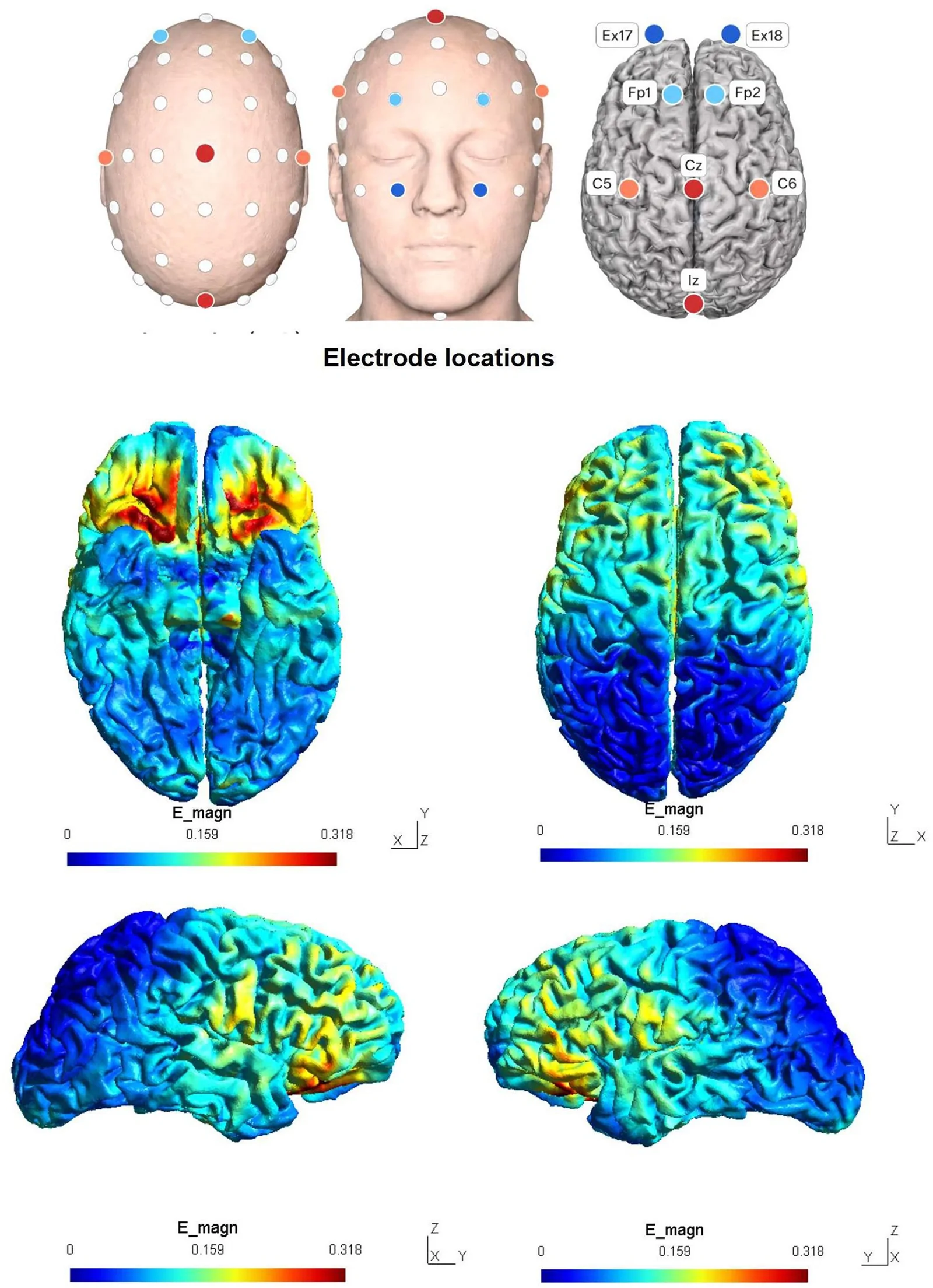
Electrode montage and electric-field modeling for the OFC-oriented theta-tACS protocol. Panel (a) shows the electrode locations from different views. Panel (b) shows the SimNIBS-modeled electric-field magnitude from inferior, superior, left lateral, and right lateral views. Warmer colors indicate greater electric-field magnitude.

Consistent with the individualized stimulation rationale, the stimulation frequency was set to each participant’s ITF rather than to a fixed theta frequency. Stimulation was delivered with eyes open for 30 min at each participant’s ITF. An independent samples *t*-test revealed no significant difference in ITF between the two groups [real: 4.88 ± 1.25 Hz, sham: 4.65 ± 1.00 Hz, t_(50)_ = .78, *P* = .424; Figure S1 (see online supplementary material for a color version of this figure) for distribution details]. All participants underwent a 30-s pulse of random noise stimulation to familiarize themselves with the skin sensations induced by tACS. The stimulation lasted 30 min, with the current ramping up to 1.8 mA over the first 10 s. In the sham group, stimulation was discontinued shortly after initiation and reintroduced for the final 10 s to maintain blinding and reduce awareness of group allocation.

### EEG recording and preprocessing

EEG recording and preprocessing followed our previous work (Huang *et al*. 2025). EEG was recorded at 1000 Hz using a 64-channel cap based on the extended 10–20 system (NeuroScan, Sterling, VA, USA), with the left mastoid as the online reference and AFz as ground. Electrode impedances were maintained below 5kΩ, and bipolar vertical and horizontal electrooculograms were recorded above and below the left eye and at the outer canthi. Signals were online band-pass filtered at 0.01–100 Hz. Offline preprocessing was performed in MATLAB using EEGLAB. Data were re-referenced to the averaged mastoids, down-sampled to 500 Hz, band-pass filtered at 0.1–100 Hz, and notch-filtered at 50 Hz. Artifact-related independent components were identified using ICLabel with visual inspection and removed. Remaining segments exceeding ±100 μV were rejected.

Task EEG data were segmented from −1000 to 2000 ms relative to stimulus onset and baseline-corrected using the −200 to 0 ms interval. Rejection rates were calculated for each participant, time point, and task condition as the number of rejected epochs divided by the original number of epochs; condition-specific rates are reported in Table S4 (see online supplementary material for a color version of this table). Individualized theta frequency was estimated from 5-min eyes-open resting-state EEG segmented into 1-s epochs. A Hamming-windowed fast Fourier transform with zero-padding and 0.1-Hz resolution was used to calculate mean power across Fz, FCz, Cz, and CPz, with ITF defined as the frequency showing maximal power within 4–8 Hz.

### Behavioral analysis

The LDI was calculated as LDI = P(“new” | lure) − P(“new” | target) (Yassa *et al*. 2010, 2011, Leal *et al*. 2014, Zheng *et al*. 2019). This index corrects for general response bias toward rejecting items as “new.” In the present study, LDI was used as a behavioral index of mnemonic discrimination, which is commonly interpreted as a behavioral proxy related to pattern separation rather than a direct neural measure of hippocampal pattern separation.

Behavioral data were analyzed using IBM SPSS Statistics (Version 22.0, IBM, Armonk, NY, USA). For emotional ratings and LDI, repeated-measures ANOVAs were conducted with valence (neutral vs. positive) and time (pre-stimulation vs. post-stimulation) as within-subject factors and stimulation group (real vs. sham) as a between-subjects factor. Bonferroni corrections were applied to multiple comparisons and simple-effects analyses, and Greenhouse–Geisser corrections were used when sphericity was violated. ANOVA effect sizes were reported as partial eta squared (ηp²). For within-group pre–post LDI comparisons, Cohen’s dz was calculated as the mean within-participant difference divided by the standard deviation of the difference scores (Lakens 2013, Goulet-Pelletier and Cousineau 2018).

### EEG analysis

During encoding, the LPP was quantified as the mean amplitude across Cz, C1, C2, CPz, CP1, and CP2 within 600–2000 ms after stimulus onset. During retrieval, the contrast-based old/new effect was evaluated using condition-specific mean amplitudes for correctly recognized targets and correctly rejected lures (Curran *et al*. 2006, Wolff *et al*. 2014, Chow *et al*. 2021). Amplitudes were extracted across Cz, C1, C2, CPz, CP1, CP2, Pz, P1, and P2 within early (250–400 ms) and late (500–1000 ms) windows (Ventura-Bort *et al*. 2021). Separate group × time × valence × trial type mixed-design ANOVAs were conducted for each window, with effects involving trial type indexing target–lure ERP differentiation. Bonferroni corrections were applied to follow-up comparisons.

Trial-level generalized linear mixed-effects models (GLMMs) with a binomial distribution and logit link function were conducted to examine whether electrophysiological measures predicted successful lure discrimination. Trial-level lure discrimination accuracy was coded as correct rejection = 1 and false alarm = 0, with participant included as a random intercept. The examined electrophysiological measures included pre-stimulus theta power and phase during the −500 to 0 ms interval and single-trial retrieval ERP amplitudes during the early (250–400 ms) and late (500–1000 ms) time windows. Theta phase was represented jointly by sine and cosine components to account for its circular nature. Separate models were fitted for theta power, theta phase, early ERP amplitude, and late ERP amplitude. The ERP models included ERP amplitude, time, stimulation group, valence, and the time × stimulation group interaction as fixed effects. The theta-phase models additionally examined whether the phase–behavior association was moderated by time and stimulation group. Wald χ^2^ tests were used to evaluate fixed effects. The ERP analyses followed recent trial-level ERP-behavior modeling approaches for binary behavioral outcomes (Liu *et al*. 2024).

EEG epoch rejection rates were analyzed using a mixed repeated-measures ANOVA with time and task condition as within-subject factors and stimulation group as a between-subjects factor. The Greenhouse–Geisser correction was applied when the assumption of sphericity was violated. Additional exploratory EEG analyses were conducted to examine task-related theta power, eyes-open resting-state theta power, pre-stimulus theta power and phase, and the contribution of encoding-stage LPP to retrieval-stage ERP amplitudes. Theta-band measures were extracted from the same frontal-central electrodes used for ITF estimation (Fz, FCz, Cz, and CPz). For task-related theta power, the mean 4–8 Hz power was extracted within the 0–1000 ms post-stimulus window using −500 to 0 ms as the baseline. Resting-state EEG data were not available for all participants at both time points. Pre-stimulation eyes-open resting-state EEG was available for 61 participants, whereas post-stimulation eyes-open resting-state EEG was available for 34 participants. Therefore, the pre–post resting-state theta analysis was conducted only in participants with paired pre- and post-stimulation resting-state EEG data (*n* = 34; real group: *n* = 20; sham group: *n* = 14).

### Questionnaires and manipulation checks

Depressive symptoms, momentary positive and negative affect, and working memory were assessed using the BDI-II, PANAS, and forward and backward Digit Span Test (DST-f and DST-b), respectively (Watson *et al*. 1988, Beck *et al*. 1996, Wechsler 1999). BDI-II scores ≥13 indicated elevated depressive symptoms (Yang *et al*. 2021). The AEQ assessed eight common tACS-related adverse effects on a 0–4 scale at the 15th minute of stimulation and immediately afterward (Clancy *et al*. 2018). Blinding was assessed by asking participants whether they believed they had received real stimulation. Blinding was successful, as the proportion of participants who believed they had received real stimulation did not differ between the real and sham groups (81.8% vs. 71.4%), χ² = 0.925, *P* = .336.

## Results

### Effects of OFC-oriented theta-tACS on behavioral performance

A three-way mixed-design ANOVA revealed a significant time × group interaction for LDI (*F*(1,59) = 4.686, *P* = .034, ηp² = .074; see Fig. 4). Post hoc analyses showed that only the real stimulation group showed a significant increase in LDI after OFC-oriented individualized theta-tACS (pre-stimulation: 0.67 ± 0.02; post-stimulation: 0.71 ± 0.02, *P* = .032, Cohen’s dz = 0.39), whereas the sham group showed no significant pre–post change (pre-stimulation: 0.70 ± 0.02; post-stimulation: 0.68 ± 0.02, *P* = .364, Cohen’s dz = −0.17; see Fig. 4). No significant main effects or other interactions were observed, including time (*F*(1,59) = 0.672, *P* = .416), valence (*F*(1,59) = 0.203, *P* = .654), valence × group (*F*(1,59) = 1.094, *P* = .300), time × valence (*F*(1,59) = 0.388, *P* = .536), and time × valence × group (*F*(1,59) = 0.004, *P* = .949).

**Figure 4:**
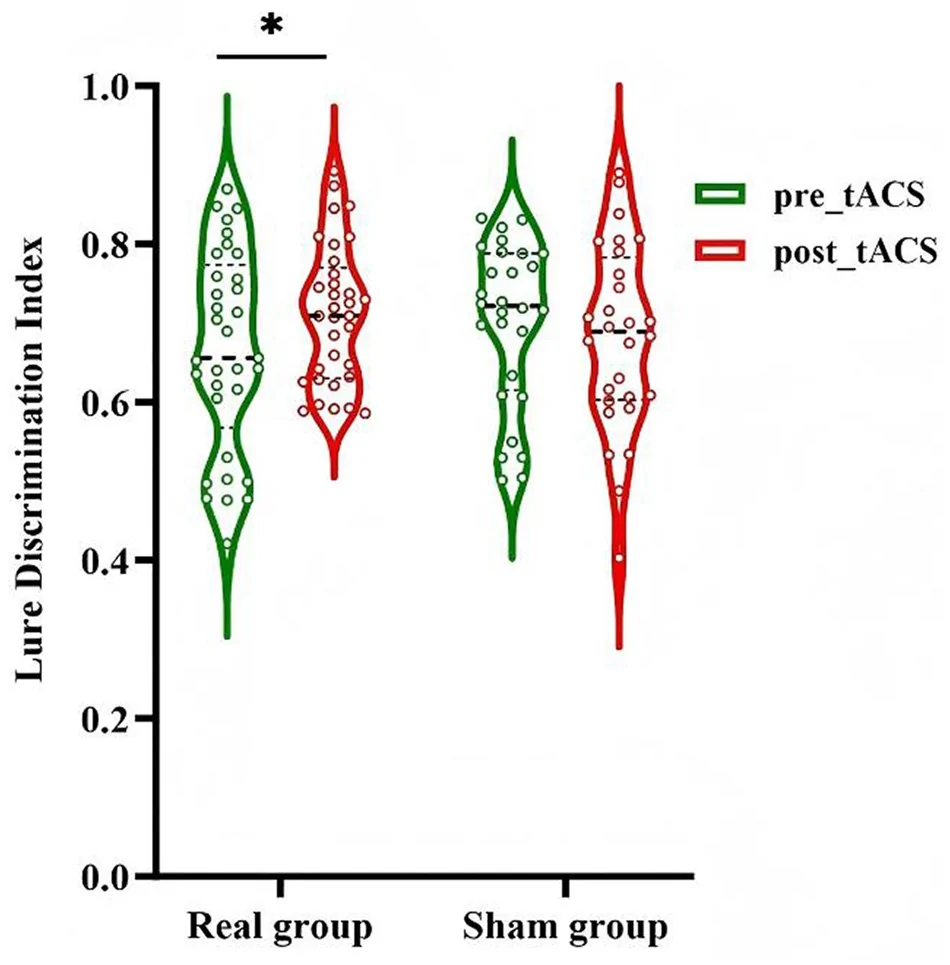
The pre-post stimulation changes of lure discrimination index (LDI) during MDT for the real and sham group. A significant increase in LDI was observed in the real stimulation group after 30 min of tACS, but not in the sham group. MDT, mnemonic discrimination task; tACS, transcranial alternating current stimulation;*P < .05.

A three-way mixed-design ANOVA revealed significant main effects of time (*F*(1,59) = 4.012, *P* = .050, ηp² = .064) and valence (*F*(1,59) = 127.612, *P* < .001, ηp² = .684) on participants’ emotional ratings. However, no significant interactions were found for time × group (*F*(1,59) = 0.118, *P* = .733), valence × group (*F*(1,59) = 0.339, *P* = .562), time × valence (*F*(1,59) = 2.216, *P* = .142), or time × group × valence (*F*(1,59) = 0.002, *P* = .964).

### Effects of individualized theta-tACS on encoding LPP and retrieval-related ERP responses

#### Encoding phase

To examine encoding-related ERP responses, a three-way mixed-design ANOVA was conducted on LPP amplitudes. A significant group × time × valence interaction was observed (*F*(1,55) = 8.209, *P* = .006, ηp² = .122), indicating that theta-tACS selectively modulated LPP amplitudes as a function of emotional valence (Fig. 5). Post hoc analysis showed a significant time × group interaction in the positive condition (*F*(1,55) = 4.233, *P* = .044, ηp² = .067). Specifically, the real group exhibited a significant increase in LPP amplitude after theta-tACS (pre-stimulation: –1.28 ± 0.56 µV vs. post-stimulation: 0.70 ± 0.44 µV, *P* = .004), whereas the sham group showed no significant change (pre-stimulation: 0.26 ± 0.61 µV vs. post-stimulation: 0.22 ± 0.47 µV, *P* = .956). Such time × group interaction was not shown in neutral trials (*F*(1,55) = 0.405, *P* = .527). No other significant main effect or interaction effect was found (all *P* > .05).

**Figure 5:**
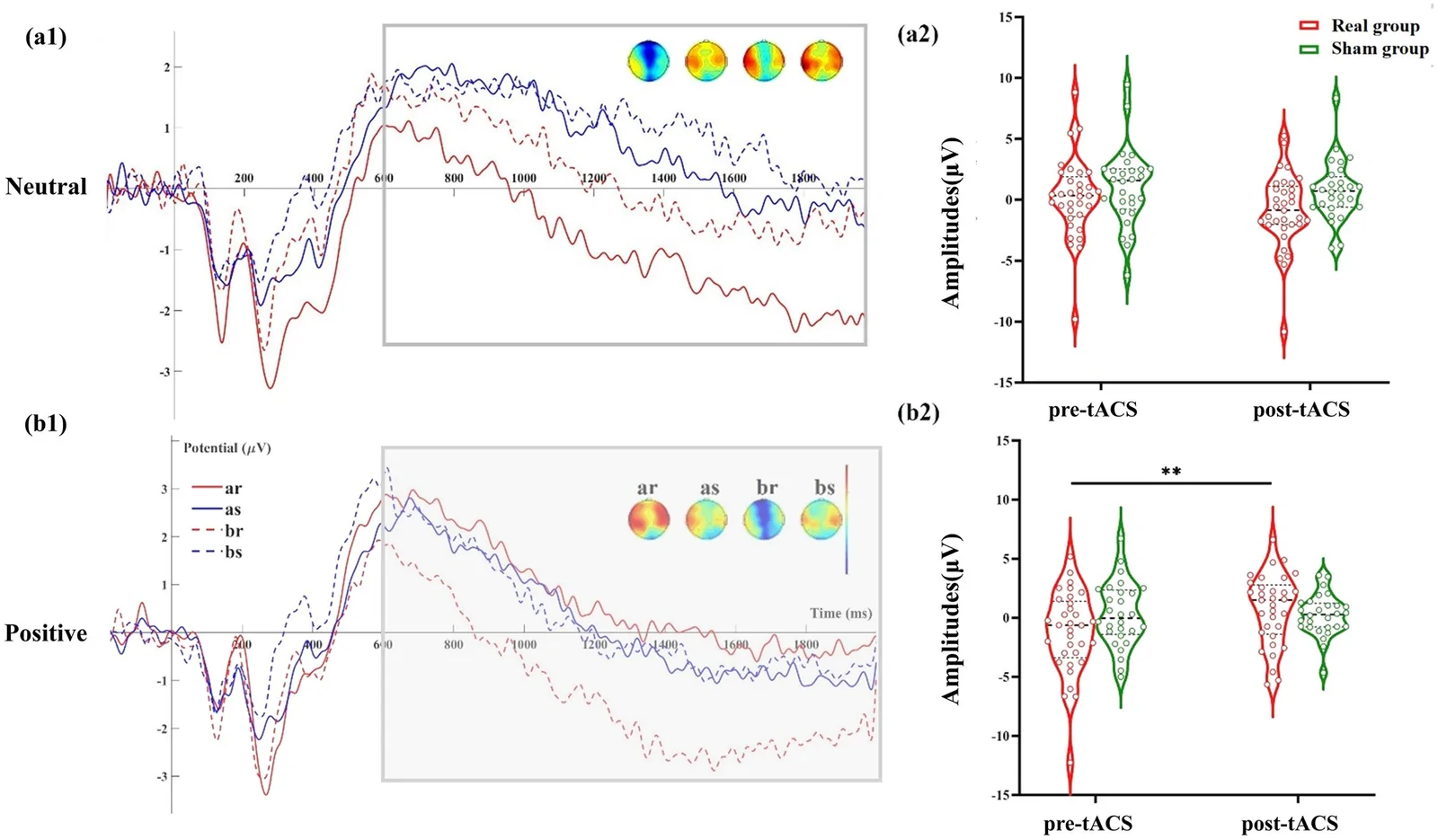
Individualized theta-tACS modulated LPP amplitudes during the encoding phase of the MDT. (a1, b1) Waveforms and corresponding topographies of the LPP component at the ROI (Cz, C1, C2, CPz, CP1, CP2) in the neutral (a1) and positive (b1) conditions, measured before (dashed lines) and after (solid lines) individualized theta-tACS and sham stimulation. Real-stimulation waveforms are labeled ar and br, whereas sham-stimulation waveforms are labeled as and bs. Gray boxes indicate the analysis window for LPP (600–2000 ms), with the shaded area in (b1) denoting a significant effect. (a2, b2) Violin plots showing individual and group-level distributions of LPP amplitudes across conditions and time points. tACS, transcranial alternating current stimulation; MDT, mnemonic discrimination task; LPP, late positive potential; ROI, region of interest; ar, after real stimulation, as, after sham stimulation; br, before real stimulation; bs, before sham stimulation; **P < .01.

#### Retrieval phase

For early retrieval-related ERP amplitudes within the 250–400 ms time window, a four-way mixed-design ANOVA revealed a significant valence × group × time interaction (*F*(1,55) = 4.186, *P* = .046, ηp² = .071), indicating that theta-tACS modulated overall early retrieval-related ERP amplitudes as a function of emotional valence (Fig. 6). Because this interaction did not involve trial type, it reflected modulation of ERP amplitudes averaged across target and lure trials rather than direct modulation of target–lure ERP differentiation. Follow-up analyses showed that, in the positive condition, the real stimulation group exhibited a significant pre–post change in mean early retrieval-related ERP amplitude following theta-tACS (pre-stimulation: 0.91 ± 0.49 µV vs. post-stimulation: −0.74 ± 0.54 µV, *P* = .003), whereas the sham group showed no significant change (pre-stimulation: 0.87 ± 0.52 µV vs. post-stimulation: 0.45 ± 0.56 µV, *P* = .461). In the neutral condition, no significant pre–post changes in mean early retrieval-related ERP amplitude were observed for either the real stimulation group (pre-stimulation: −0.49 ± 0.62 µV vs. post-stimulation: −0.21 ± 0.61 µV, *P* = .703) or the sham group (pre-stimulation: −1.01 ± 0.64 µV vs. post-stimulation: −0.10 ± 0.66 µV, *P* = .157).

**Figure 6:**
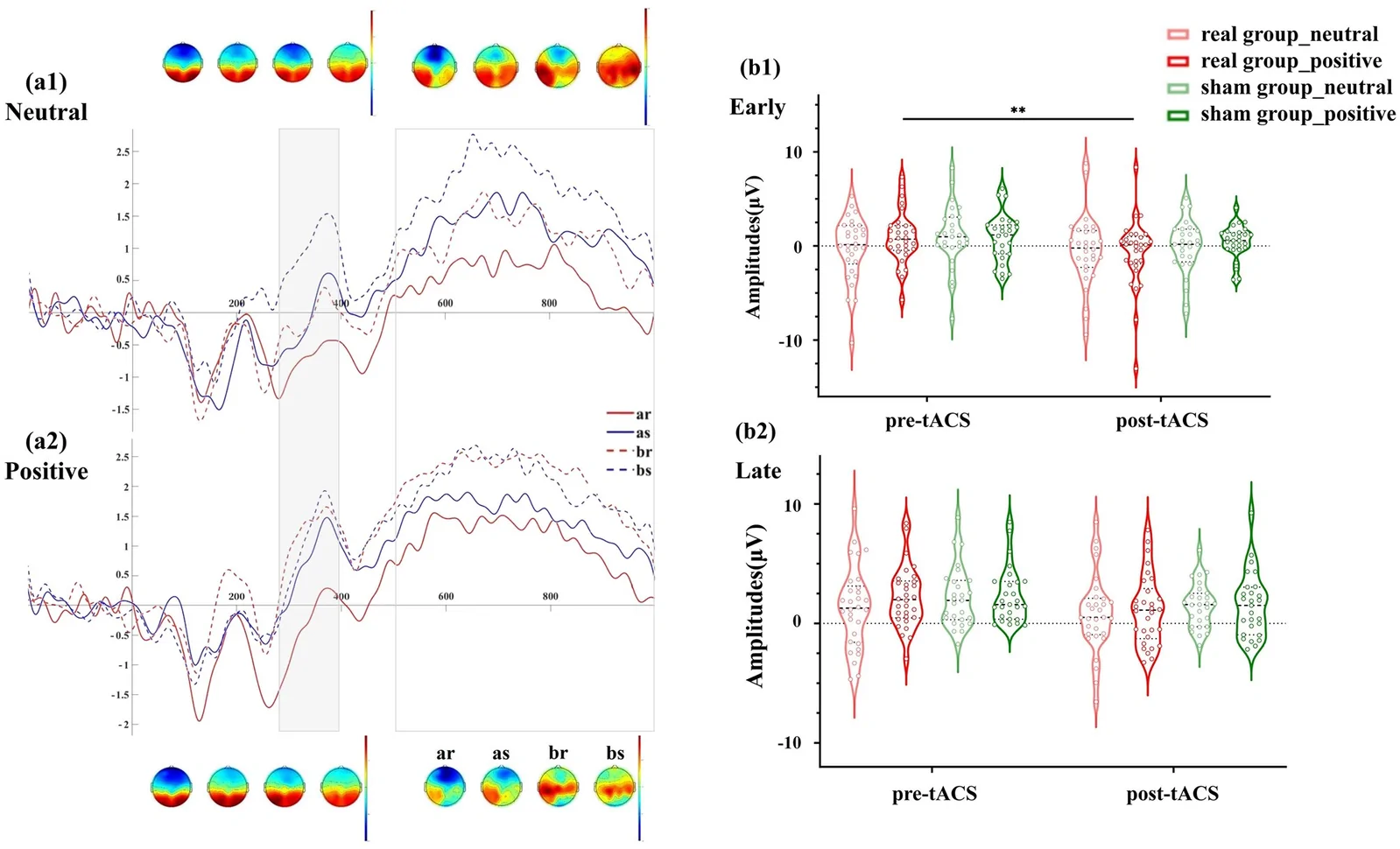
Individualized theta-tACS modulated early retrieval-related ERP amplitudes during the retrieval phase of the MDT. (a1, a2) ERP waveforms and corresponding topographies within the early (250–400 ms, left gray box) and late (500–1000 ms, right gray box) retrieval time windows at the predefined ROI (Cz, C1, C2, CPz, CP1, CP2, Pz, P1, and P2) for neutral (a1) and positive (a2) conditions, comparing conditions before and after stimulation in the real and sham groups. Before-stimulation waveforms are shown as dashed lines (br and bs), whereas after-stimulation waveforms are shown as solid lines (ar and as). Real-stimulation waveforms are labeled ar and br, whereas sham-stimulation waveforms are labeled as and bs.The shaded area in the positive condition denotes the early retrieval time window in which a significant group × time × valence effect was observed. This effect reflects overall retrieval-related ERP amplitudes averaged across target and lure trials rather than direct modulation of target–lure differentiation. (b1, b2) Violin plots showing distributions of early (b1) and late (b2) retrieval-related ERP amplitudes across valence conditions, groups, and time points. tACS, transcranial alternating current stimulation; MDT, mnemonic discrimination task; ERP, event-related potential; ar, after real stimulation; as, after sham stimulation; br, before real stimulation; bs, before sham stimulation. **P < .01.

The same four-way ANOVA also revealed a significant main effect of time (*F*_(1,55)_ = 4.774, *P* = .033, ηp² = .080) and a significant valence × trial type interaction (*F*_(1,55)_ = 6.196, *P* = .016, ηp² = .101). Post hoc analyses indicated a significant early old/new effect in the neutral condition, with larger positive amplitudes for target trials than lure trials (target: 0.31 ± 0.40 µV vs. lure: −0.20 ± 0.35 µV, *P* = .029). In contrast, no significant target–lure difference was observed in the positive condition (target: 0.19 ± 0.37 µV vs. lure: 0.56 ± 0.32 µV, *P* = .194). No other significant main effects or interactions, including the group × time × valence × trial type interaction, were observed (all *P* > .05).

For late retrieval-related ERP amplitudes within the 500–1000 ms time window, a separate four-way mixed-design ANOVA revealed no significant tACS-related modulation effects. The analysis revealed a significant main effect of time (*F*_(1,55)_ = 4.521, *P* = .038, ηp² = .076) and a significant valence × trial type interaction (*F*_(1,55)_ = 10.228, *P* = .002, ηp² = .157). Post hoc analyses showed a significant late old/new effect in the neutral condition, with higher amplitudes for target trials than lure trials (target: 2.01 ± 0.39 µV vs. lure: 0.92 ± 0.29 µV, *P* = .001), whereas no significant target–lure difference was observed in the positive condition (target: 1.58 ± 0.38 µV vs. lure: 2.00 ± 0.30 µV, *P* = .248). No other significant main effects or interactions were observed (all *P* > .05). As an exploratory sensitivity analysis, we further examined whether the retrieval-stage ERP findings remained evident after accounting for encoding-stage LPP amplitudes; the complete analysis and results are reported in Supplementary Analysis S1.

### Psychological assessment

The real and sham groups did not differ in demographic characteristics or baseline psychological measures (all *P* > .05; Table 1). Three-way repeated-measures ANOVAs revealed significant effects of time, dimension, and time × dimension on PANAS scores (all *P* < .001), but neither the time × group interaction, *F*(1,59) = 0.956, *P* = .332, nor the time × group × dimension interaction, *F*(1,59) = 0.101, *P* = .752, was significant. For the DST, significant main effects of time and dimension (both *P* < .001) were accompanied by a time × group interaction, *F*(1,59) = 7.219, *P* = .009, ηp² = .109. Digit span scores increased in the sham group (7.05 ± 0.25 to 7.84 ± 0.21, *P* < .001) but not in the real stimulation group (7.42 ± 0.23 to 7.64 ± 0.19, *P* = .148). No other group-related interactions were significant.

**Table 1:** Baseline characteristics and pre-post changes in MDT-related measures between sham and real stimulation groups.

Measures		Sham	Real	*t*/χ^2^	*P*
		Mean (SD)	Mean (SD)		
Demographic data					
Age		20.46 ± 1.47	20.49 ± 1.62	−0.051	.959
Sex, male: female		10:18	9:24	0.503	.478
Education, yr		14.04 ± 1.55	14.00 ± 1.54	0.090	.929
Pre-tACS psychometric score					
BDI-II		24.25 ± 6.47	27.85 ± 9.92	−1.70	.095
SAS		55.54 ± 12.18	59.76 ± 12.69	−1.319	.192
PANAS-PA		24.50 ± 3.92	23.85 ± 5.22	0.543	.589
PANAS-NA		33.14 ± 9.98	36.85 ± 11.16	−1.356	.180
DST-f		8.75 ± 0.93	8.825 ± 1.26	−0.237	.814
DST-b		5.36 ± 2.06	6.03 ± 1.83	−1.325	.181
MDT performance				Group × time interaction/*F*	
Emotional rating_ neutral	pre_tACS	5.11 ± 0.71	4.91 ± 5.11	0.118	.733
	post_tACS	5.29 ± 0.77	5.13 ± 0.51		
Emotional rating_positive	pre_tACS	6.04 ± 0.77	5.75 ± 0.88		
	post_tACS	6.11 ± 0.83	5.87 ± 0.83		
LDI_neutral	pre_tACS	0.69 ± 0.12	0.67 ± 0.14	4.686	.034*
	post_tACS	0.67 ± 0.13	0.71 ± 0.12		
LDI_positive	pre_tACS	0.71 ± 0.11	0.66 ± 0.14		
	post_tACS	0.69 ± 0.13	0.71 ± 0.10		

BDI-II, Beck Depression Inventory-II; SAS, Self-Rating Anxiety Scale; PANAS-PA, Positive Affect subscale of the Positive and Negative Affect Schedule; PANAS-NA, Negative Affect subscale of the Positive and Negative Affect Schedule; DST-f, Digit Span Test (forward); DST-b, Digit Span Test (backward); MDT, Mnemonic Discrimination Task; LDI, Lure Discrimination Index; tACS, transcranial alternating current stimulation; SD, standard deviation.

* *P* < .05.

### Trial-level ERP–behavior associations

To further examine whether ERP activity predicted successful lure discrimination at the single-trial level, GLMMs) were conducted using trial-level lure discrimination accuracy as the dependent variable. Neither early nor late retrieval ERP amplitude significantly predicted trial-level lure discrimination accuracy, early: χ²(1) = 2.108, *P* = .147; late: χ²(1) = 1.593, *P* = .207. A significant main effect of time was observed in both models, indicating higher lure discrimination accuracy at post-stimulation, whereas no other effects were significant (all *P* > .05). Thus, retrieval ERP amplitudes were not reliable single-trial predictors of successful lure discrimination.

### Exploratory participant-level correlations

To complement the trial-level analyses, exploratory participant-level correlation analyses were conducted within the real stimulation group. Specifically, change scores (post-stimulation minus pre-stimulation) in behavioral measures, including positive emotional ratings during the MDT and LDI, were correlated with changes in encoding-related LPP amplitudes. No significant correlations were found between changes in positive emotional ratings and changes in LPP amplitude (*r *=* −*0.13, *P *= .495), or between changes in LDI and the enhancement of LPP amplitude (*r *= 0.007, *P *= .973).

### Exploratory theta-related analyses

Additional exploratory theta-related analyses were conducted to examine potential oscillatory mechanisms. Task-related theta-band power did not show a significant time × stimulation group interaction, *F*(1,55) = 2.66, *P* = .108, indicating that pre-to-post changes in task-related theta power did not differ significantly between the real and sham stimulation groups. Resting-state theta power showed a significant main effect of time, *F*(1,32) = 6.73, *P* = .014, but the time × stimulation group interaction was not significant, *F*(1,32) = 0.42, *P* = .522.

At the trial level, pre-stimulus theta power did not significantly predict lure correct rejection, χ²(1) = 0.23, *P* = .631. In contrast, circular mean theta phase during the −500 to 0 ms pre-stimulus interval significantly improved the prediction of lure correct rejection, χ²(2) = 8.03, *P* = .018. However, the phase × time interaction was not significant, χ²(2) = 1.95, *P* = .377, and the phase × stimulation group × time interaction was also not significant, χ²(2) = 2.20, *P* = .333. Thus, pre-stimulus theta phase was associated with trial-level lure discrimination, but this association did not significantly change from pre- to post-stimulation, and the extent of change did not differ between the real and sham stimulation groups. Therefore, the present findings do not provide evidence that theta-tACS altered the theta phase − behavior association.

### Safety evaluation of tACS

We evaluated the safety of the current tACS protocol based on the responses on AEQ. AEQ ratings were generally low in both groups. Neither the main effect of stimulation group nor any group-related interactions were significant (all *P* > .05), indicating comparable tolerability between real and sham stimulation. Detailed results are provided in the Supplementary Material.

### EEG epoch rejection quality-control analysis

EEG epoch rejection was comparable between the real and sham stimulation groups. The mixed repeated-measures ANOVA showed no significant main effect of stimulation group, *F*(1, 55) = 0.25, *P* = .622, and no significant group-related interactions, including time × stimulation group, *F*(1, 55) = 3.06, *P* = .086, condition × stimulation group, *F*(2.31, 126.87) = 0.74, *P* = .496, and time × condition × stimulation group, *F*(2.25, 123.83) = 1.47, *P* = .233. Group-level rejection rates for each time point and task condition are provided in Table S4 (see online supplementary material for a color version of this table).

## Discussion

This double-blind, sham-controlled study examined the effects of OFC-oriented individualized theta-tACS on mnemonic discrimination and ERP activity in individuals with elevated depressive symptoms. Real stimulation was associated with increased LDI, enhanced LPP amplitudes during positive encoding, and altered early retrieval-related ERP activity. These findings suggest that individualized theta-tACS may influence both behavioral discrimination performance and neural responses to positive information. However, because the behavioral LDI effect was not significantly moderated by valence and the ERP-behavior associations were not robust, the behavioral and electrophysiological findings should be interpreted as complementary but not directly coupled evidence.

The behavioral findings suggest that OFC-oriented individualized theta-tACS may influence mnemonic discrimination. The real stimulation group showed increased LDI scores after stimulation, whereas the sham group did not. Because LDI indexes the ability to reject similar lure items while correcting for general “new” response bias, this finding suggests improved discrimination of overlapping mnemonic information. However, the absence of a significant time × group × valence interaction indicates that this behavioral effect was not specific to positive stimuli. In addition, theta-tACS did not produce a group-specific modulation of emotional ratings, suggesting that a single session of stimulation may influence mnemonic discrimination without producing immediate changes in subjective emotional experience.

The ERP findings provide complementary evidence that individualized theta-tACS may modulate neural processes involved in positive memory encoding. During encoding, the real stimulation group showed increased LPP amplitudes for positive stimuli after stimulation, whereas the sham group showed no comparable change. The LPP is widely considered an ERP component sensitive to emotional salience, motivational relevance, and memory encoding processes. Larger LPP amplitudes to emotional stimuli have been interpreted as reflecting enhanced allocation of attentional and encoding resources to motivationally salient information. In the present study, the selective increase in LPP amplitudes under the positive condition may therefore suggest that OFC-oriented individualized theta-tACS influenced the neural processing of positive mnemonic information during encoding.

During retrieval, individualized theta-tACS modulated overall early retrieval-related ERP amplitudes as a function of emotional valence, whereas no comparable stimulation-related modulation was observed in the late time window. Importantly, the significant early group × time × valence interaction did not involve trial type and therefore should be interpreted as modulation of retrieval-related ERP activity averaged across target and lure trials, rather than as direct modulation of the target–lure old/new effect. In addition, significant valence × trial type interactions were observed in both the early and late time windows, indicating that target–lure ERP differentiation varied between neutral and positive conditions. Specifically, significant target–lure differences were observed in the neutral condition but not in the positive condition. Thus, the findings suggest that individualized theta-tACS influenced early retrieval-related neural activity, whereas the observed target–lure differentiation was primarily related to emotional valence. Moreover, trial-level ERP-behavior analyses did not show that ERP amplitudes reliably predicted successful lure discrimination, indicating that the ERP and behavioral findings should not be interpreted as directly coupled.

The theta-related exploratory analyses provide only limited support for a theta-phase-behavior account. The interpretation of the retrieval ERP findings is broadly consistent with the SPEAR model (Hasselmo *et al*. 2002, Hasselmo 2025), which proposes that encoding and retrieval are supported by distinct phases of the theta cycle. In the present study, pre-stimulus theta phase during the −500 to 0 ms interval was associated with trial-level lure correct rejection, whereas pre-stimulus theta power was not. However, the phase-behavior association was not significantly modulated by time or by the phase × stimulation group × time interaction. Moreover, exploratory analyses did not provide evidence for real stimulation-specific modulation of task-related theta power or post-stimulation resting-state theta power. Therefore, the present data are compatible with, but do not demonstrate, a theta-mediated tACS mechanism.

The observed effects may also involve OFC-related modulation of broader memory-related networks. As outlined in the Introduction, the OFC may serve as a connected prefrontal node within hippocampal–amygdala–prefrontal circuits rather than as an isolated stimulation locus. In the present task, participants needed to reject similar but non-identical lure items rather than endorse them as previously seen targets, a demand that may rely on OFC-supported contextual evaluation and flexible memory-based decision-making. Thus, individualized theta-tACS delivered with an OFC-oriented montage may have perturbed OFC-centered network dynamics relevant to ambiguous or overlapping mnemonic information. This interpretation is consistent with evidence linking the OFC to contextual and value-related representations, cognitive-map-like organization, and interactions with medial temporal lobe systems (Young and Shapiro 2011, Wikenheiser and Schoenbaum 2016, Knudsen and Wallis 2022, Zheng *et al*. 2022, Costa *et al*. 2023, Qiu *et al*. 2024). However, given the diffuse nature and limited spatial focality of tACS-induced electric fields, the present findings should not be interpreted as evidence for focal, selective, or exclusive modulation of the OFC.

The HD-tACS protocol was well tolerated, with only mild and transient side effects, no severe discomfort, and no significant group differences, consistent with previous tACS safety findings (Matsumoto and Ugawa 2017). Burning was the highest-rated sensation, whereas phosphenes remained mild. Phosphenes are commonly reported during tACS and may arise from retinal or visual-cortical stimulation (Schwiedrzik 2009, Kar and Krekelberg 2012), potentially depending on the stimulation montage and current distribution.

Several limitations should be noted. First, although the present findings suggest that individualized theta-tACS may modulate behavioral and electrophysiological indices related to mnemonic discrimination, the oscillatory mechanism remains unresolved. Exploratory theta analyses did not provide evidence for real stimulation-specific modulation of task-related theta power, post-stimulation resting-state theta power, or the theta phase-behavior association. Although pre-stimulus theta phase was associated with trial-level lure discrimination, this association was not significantly modulated by stimulation group or time. Therefore, the present EEG data are compatible with, but do not demonstrate, a theta-mediated tACS mechanism. Future studies should combine tACS with EEG or MEG protocols optimized for assessing stimulation-induced oscillatory changes, including concurrent recording, active artifact-control procedures, and active frequency-control conditions. Second, the absence of an active frequency-control condition, such as alpha-tACS, limits our ability to attribute the observed effects specifically to theta-frequency stimulation. Therefore, future studies should include active stimulation conditions at non-theta frequencies to determine whether the observed behavioral and ERP effects are specific to individualized theta-frequency stimulation. Third, because stimulation, encoding, and retrieval were administered sequentially within the same session, the present design cannot determine whether the observed effects arose primarily from modulation of encoding-related or retrieval-related processes. Although the exploratory covariate analysis provides indirect information, statistical adjustment cannot establish stage-specific causality. Future studies could directly distinguish these possibilities by comparing stimulation administered before encoding with stimulation administered after encoding but before retrieval, together with matched sham conditions. A stronger effect following pre-encoding stimulation would be more consistent with an encoding-related contribution, whereas an effect following post-encoding stimulation would be more consistent with a retrieval-related contribution. Thus, the retrieval ERP findings should be interpreted cautiously and should not be taken as evidence for a retrieval-specific mechanism. Fourth, the pre-stimulation and post-stimulation sessions were administered in a fixed order rather than counterbalanced, which may introduce practice or order effects despite the use of different stimulus sets. Finally, this study used only a single stimulation session and did not include follow-up assessments; therefore, the durability and clinical relevance of the observed effects remain to be established in future repeated-session studies, particularly in clinically diagnosed patients with major depressive disorder.

## Conclusion

The findings provide preliminary evidence that a single session of OFC-oriented individualized theta-tACS was associated with improved mnemonic discrimination and modulation of positive encoding-related LPP and valence-dependent early retrieval ERP activity in individuals with elevated depressive symptoms. Studies using active frequency-control conditions, counterbalanced designs, and repeated sessions are needed to establish the frequency specificity, durability, and mechanisms of these effects.

## Supplementary Material

nsag079_Supplementary_Data

## Data Availability

The data supporting the findings of this study are available from the corresponding authors upon reasonable request but are not publicly accessible to protect participant privacy.
