# Supplementary material for: Individualized theta transcranial alternating current stimulation modulates mnemonic discrimination and event-related potential responses to positive information in individuals with elevated depressive symptoms
Source: Soc Cogn Affect Neurosci. 2026 Oct 16;21(1):nsag079. doi: 10.1093/scan/nsag079 (PMC13695148; doi:10.1093/scan/nsag079)
Supplement: Supplementary file 1 [file nsag079_supplementary_data.docx]

**Supplementary Material**

|  | Set A | | Set B | | Image set main effect | | Valence main effect | | Interactions | |
| --- | --- | --- | --- | --- | --- | --- | --- | --- | --- | --- |
|  | Neutral | Positive | Neutral | Positive | F | P | F | P | F | P |
| Similarity | 5.47±0.98 | 5.69±1.29 | 5.39±0.98 | 5.74±1.27 | 0.003 | 0.959 | 2.330 | 0.129 | 0.116 | 0.734 |
| Valence | 5.75±0.10 | 6.60±0.09 | 5.58±0.09 | 6.55±0.09 | 1.393 | 0.240 | 94.597 | <0.001*** | 0.395 | 0.531 |
| Arousal | 4.79±0.09 | 5.59±0.09 | 4.61±0.09 | 5.77±0.09 | <0.001 | 0.996 | 114.624 | <0.001*** | 3.804 | 0.053 |
| Salience | 5.92±0.11 | 6.52±0.10 | 5.74±0.11 | 6.48±0.10 | 1.027 | 0.312 | 39.926 | <0.001*** | 0.389 | 0.534 |

**Table S1:** The table presents four psychological attributes (similarity, valence, arousal, and salience) for neutral and positive images in image sets A and B during the encoding phase (mean ± SD) and the statistical results. Repeated-measures ANOVA showed that the main effects of image set and the interaction effects were not significant for any of the four attributes.（* p < 0.05；** p < 0.01；*** p < 0.001）.

| Visual attributes | Set A | | | | Set B | | | |
| --- | --- | --- | --- | --- | --- | --- | --- | --- |
|  | Neutral | | Positive | | Neutral | | Positive | |
|  | target | lure | target | lure | target | lure | target | lure |
| Similarity | 5.21±1.29 | 5.47±1.00 | 5.3±1.57 | 5.68±1.34 | 4.83±0.99 | 5.38±1.00 | 5.44±1.41 | 5.70±1.31 |
| Height | 351.3±51.31 | 378.03±51.60 | 368.38±54.84 | 383.51±53.64 | 369.85±45.36 | 363.66±46.36 | 350.54±47.10 | 379.09±57.05 |
| Width | 412.46±39.43 | 405.86±45.34 | 394.23±53.97 | 413.60±58.58 | 399.54±44.48 | 415.80±34.14 | 415.62±52.03 | 398.43±50.36 |
| Brightness | 117.96±45.51 | 113.26±37.05 | 125.82±54.08 | 122.89±45.03 | 138.87±44.43 | 119.35±38.80 | 141.82±29.39 | 117.42±37.51 |
| Contrast | 60.03±17.51 | 63.26±15.27 | 56.54±15.63 | 57.32±12.49 | 50.42±19.66 | 59.14±17.92 | 58.71±19.94 | 60.03±14.15 |
| LABL | 8.79±7.38 | 4.34±5.65 | 8.09±4.49 | 4.41±4.64 | 11.14±12.36 | 4.15±4.00 | 10.54±8.38 | 4.43±5.08 |
| LABA | 0.80±1.95 | 0.30±0.58 | 1.09±2.32 | 0.82±1.88 | 0.59±1.66 | 0.04±0.36 | 0.20±1.40 | 0.41±1.25 |
| LABB | 1.08±2.54 | 0.55±1.20 | 1.50±3.20 | 1.09±2.24 | -0.17±2.04 | -0.06±0.93 | 1.82±2.07 | 1.00±1.76 |
| Entropy | 7.02±0.56 | 7.08±0.78 | 6.88±0.84 | 7.11±0.55 | 7.04±0.70 | 7.10±0.67 | 7.17±0.46 | 7.18±0.56 |

**Table S2:** The table presents the results of similarity and six visual attribute measurements (height, width, brightness, contrast, color composition, entropy) for target and lure images in image sets A and B during the retrieval phase (means ±SD).

| Visual attributes | Image sets | | Valence | | | Type | | Sets *  Valence | | Sets *  Type | | Valence *  Type | | Sets *  Valence *type | |
| --- | --- | --- | --- | --- | --- | --- | --- | --- | --- | --- | --- | --- | --- | --- | --- |
|  | F | P | F | P | | F | P | F | P | F | P | F | P | F | P |
| Similarity | 0.160 | 0.689 | 2.429 | | 0.121 | 3.369 | 0.068 | 0.634 | 0.427 | 0.034 | 0.855 | 0.053 | 0.817 | 0.293 | 0.589 |
| Height | 0.294 | 0.589 | 0.307 | | 0.580 | 3.652 | 0.058 | 0.617 | 0.433 | 0.335 | 0.564 | 0.479 | 0.490 | 1.899 | 0.170 |
| Width | 0.011 | 0.917 | 0.143 | | 0.705 | 0.145 | 0.704 | 0.087 | 0.768 | 0.194 | 0.661 | 0.058 | 0.810 | 3.646 | 0.058 |
| Brightness | 1.991 | 0.160 | 0.485 | | 0.487 | 3.755 | 0.054 | 0.382 | 0.537 | 1.859 | 0.174 | 0.014 | 0.907 | 0.063 | 0.803 |
| Contrast | 0.730 | 0.394 | 0.001 | | 0.982 | 1.833 | 0.177 | 3.217 | 0.075 | 0.337 | 0.562 | 0.900 | 0.344 | 0.228 | 0.634 |
| LA  BL | 1.397 | 0.239 | 0.063 | | 0.802 | 28.957 | 0.001** | 0.004 | 0.948 | 1.614 | 0.206 | 0.182 | 0.670 | 0.002 | 0.968 |
| LA  BA | 3.834 | 0.052 | 0.792 | | 0.375 | 1.513 | 0.220 | 0.818 | 0.367 | 0.208 | 0.649 | 1.221 | 0.271 | 0.342 | 0.559 |
| LA  BB | 1.793 | 0.182 | 10.717 | | 0.001** | 1.810 | 0.180 | 2.892 | 0.091 | 0.035 | 0.853 | 0.446 | 0.505 | 0.733 | 0.393 |
| Entropy | 0.718 | 0.398 | 0.098 | | 0.755 | 0.838 | 0.361 | 0.670 | 0.414 | 0.202 | 0.654 | 0.049 | 0.825 | 0.277 | 0.600 |

**Table S3:** The table shows the results of repeated-measures ANOVAs on similarity and six visual attributes (height, width, brightness, contrast, color composition, entropy) for image sets A and B during the retrieval phase, with image set × valence type as the repeated-measures factors. The results indicate that images in sets A and B were matched during the retrieval phase (* p < 0.05；** p < 0.01；*** p < 0.001).

| **Group** | **Time** | **Condition** | **n** | **Rejection rate, M ± SD (%)** |
| --- | --- | --- | --- | --- |
| Real  Real  Real  Real  Real  Real  Real  Real | Pre-stimulation  Pre-stimulation  Pre-stimulation  Pre-stimulation | Encoding | 30 | 3.77 ± 4.19 |
|  |  | Retrieval-target | 30 | 4.87 ± 5.43 |
|  |  | Retrieval-lure | 30 | 4.48 ± 3.67 |
|  |  | Retrieval-foil | 30 | 4.29 ± 3.78 |
|  | Post-stimulation  Post-stimulation  Post-stimulation  Post-stimulation | Encoding | 30 | 3.53 ± 4.14 |
|  |  | Retrieval-target | 30 | 3.23 ± 4.06 |
|  |  | Retrieval-lure | 30 | 4.01 ± 3.34 |
|  |  | Retrieval-foil | 30 | 4.13 ± 4.15 |
| Sham  Sham  Sham  Sham  Sham  Sham  Sham  Sham | Pre-stimulation  Pre-stimulation  Pre-stimulation  Pre-stimulation | Encoding | 27 | 3.47 ± 4.72 |
|  |  | Retrieval-target | 27 | 2.95 ± 4.19 |
|  |  | Retrieval-lure | 27 | 4.42 ± 3.56 |
|  |  | Retrieval-foil | 27 | 3.38 ± 3.14 |
|  | Post-stimulation  Post-stimulation  Post-stimulation  Post-stimulation | Encoding | 27 | 3.68 ± 4.39 |
|  |  | Retrieval-target | 27 | 4.84 ± 8.37 |
|  |  | Retrieval-lure | 27 | 6.03 ± 7.59 |
|  |  | Retrieval-foil | 27 | 6.71 ± 7.53 |

**Table S4:** Group-level EEG epoch rejection rates by stimulation group, time point, and task condition. Rejection rate was calculated as the number of rejected epochs divided by the number of original epochs before artifact rejection and is presented as a percentage. Values are means ± standard deviations. Real stimulation group, n = 30; sham stimulation group, n = 27.

Figure S1: The ITF distributions for the real and sham stimulation groups showed no significant differences. ITF: individualized theta frequency (individualized frequency peak).

**Safety Evaluation of tACS**

We evaluated the safety of the current tACS protocol based on the responses on AEQ. AEQ ratings were generally low in both groups. Neither the main effect of stimulation group nor any group-related interactions were significant (all p > .05), indicating comparable tolerability between real and sham stimulation. Detailed results are provided in the Supplementary Material.

We evaluated the safety of the current tACS protocol based on the responses on AEQ. During tACS, participants in the sham group reported the three most intense side effects as burning (1.22 ± 0.20), fatigue (0.78 ± 0.22), and itching (0.63 ± 0.15), whereas those in the real stimulation group reported burning (1.24 ± 0.20), fatigue (1.06 ± 0.20), and phosphene (1.06 ± 0.18) as the most intense. Repeated-measures ANOVA revealed significant main effects of time (F(1,58) = 36.417, p < .001, ηp² = 0.386), AEQ item (F(7,52) = 7.859, p < .001, ηp² = 0.514), and a significant time × AEQ item interaction effect (F(7,52) = 4.352, p = .001, ηp² = 0.369). The main effect of group and other interaction effects were not significant (all p’s > .05). Post hoc analyses indicated that, after tACS, five side effects—tingling (p < .001), itching (p < .001), burning (p < .001), nervousness (p = .024), and phosphene (p = .001)—significantly decreased. However, no significant reductions in fatigue, difficulty concentrating, and headache were suggested (all p’s > .05).

**Stimulus Validation and Composition**

To minimize practice effects, two stimulus sets (Sets A and B) were used for the pre- and post-stimulation assessments in an ABBA counterbalancing order. Target–lure similarity was evaluated in an independent sample of 31 college students (16 females; mean age = 23.94 years). Participants rated the similarity of each encoding image and its corresponding lure on a 9-point scale, with 9 indicating that the images were identical. Because emotional images may be perceived as more similar than neutral images (Zheng et al., 2019), target–lure similarity was evaluated while accounting for emotional valence. The analysis revealed no significant effects of valence, F(1,147) = 2.330, p = .129, image set, F(1,147) = 0.003, p = .959, or their interaction, F(1,147) = 0.116, p = .734.

Image valence, arousal, and salience were also compared across the two sets. As expected, the main effect of valence was significant (p < .001), whereas neither image-set effects nor set-related interactions were significant (all p > .05; Supplementary Table S1). Retrieval-phase materials were further evaluated using a 2 (image set: A vs. B) × 2 (valence: neutral vs. positive) × 2 (trial type: target vs. lure) repeated-measures ANOVA. No significant image-set effects or set-related interactions were found for target–lure similarity (all p > .05; Supplementary Tables S2–S3). Visual attributes, including image size, brightness, contrast, entropy, and color composition, were calculated following Marchewka et al. (2014). These attributes were comparable across image sets, valence conditions, and image types, with no significant effects or interactions (all p > .05; Supplementary Tables S2–S3).

The stimulus pool included 197 neutral and 200 positive images selected from the Chinese Affective Picture System (Lu, Hui, & Yu-Xia, 2005). An additional 52 images (27 neutral and 25 positive) were generated using Midjourney to provide sufficient lure stimuli. In Set A, the encoding phase contained 74 images (36 neutral and 38 positive). The retrieval phase contained 88 neutral images (13 targets, 35 lures, and 40 foils), including 15 AI-generated lures, and 88 positive images with the same target–lure–foil composition, including 13 AI-generated lures. In Set B, the encoding phase contained 75 images (37 neutral and 38 positive). The retrieval phase contained 88 neutral and 88 positive images per valence condition, each comprising 13 targets, 35 lures, and 40 foils; 12 lures in each valence condition were AI-generated.

**Supplementary Analysis S1. Exploratory retrieval ERP analyses controlling for encoding-stage LPP**

To examine whether encoding-stage neural activity could account for the retrieval-stage ERP findings, we conducted exploratory sensitivity analyses in which standardized encoding-stage LPP amplitude was included as a covariate. Separate linear mixed-effects models were fitted for the early (250–400 ms) and late (500–1000 ms) retrieval windows. Each model included stimulation group, time, valence, trial type, and all factorial interactions among these variables as fixed effects, standardized encoding-stage LPP amplitude as an additional covariate, and participant as a random intercept. The models were specified as follows: retrieval ERP amplitude ~ group × time × valence × trial type + encoding LPP z score + (1 | participant). Degrees of freedom and significance tests were estimated using the Satterthwaite approximation. Because these analyses were not part of the primary analysis plan, they were treated as exploratory sensitivity analyses.

Encoding-stage LPP significantly predicted retrieval-stage ERP amplitudes in both the early retrieval window, F(1, 438.36) = 10.91, p = .001, and the late retrieval window, F(1, 415.90) = 30.29, p < .001. Thus, encoding-stage LPP was associated with subsequent retrieval-stage ERP amplitudes. After accounting for encoding-stage LPP, the group × time × valence interaction remained significant in the early retrieval window, F(1, 384.08) = 8.03, p = .005, but was not significant in the late retrieval window, F(1, 385.88) = 2.08, p = .150. Therefore, the overall early retrieval-related ERP modulation remained statistically detectable after adjustment for encoding-stage LPP. For effects involving trial type, the valence × trial type interaction did not reach significance in the early retrieval window after adjustment for encoding-stage LPP, F(1, 381.89) = 3.32, p = .069, whereas it remained significant in the late retrieval window, F(1, 382.95) = 8.94, p = .003. The group × time × valence × trial type interaction was not significant in either the early retrieval window, F(1, 381.89) = 0.40, p = .526, or the late retrieval window, F(1, 382.95) = 0.04, p = .845. Thus, the exploratory analysis provided no evidence that stimulation modulated target–lure ERP differentiation after accounting for encoding-stage LPP.

Overall, these findings indicate that encoding-stage LPP was associated with retrieval-stage ERP amplitudes and did not fully account for the overall early group × time × valence effect. However, because encoding and retrieval occurred sequentially within the same experimental session, statistical adjustment cannot establish that this effect arose specifically during retrieval.
